# Telbivudine can safely reduce mother-to-child transmission in chronic hepatitis B women after 12 weeks of gestation

**DOI:** 10.1186/s12879-019-4250-6

**Published:** 2019-07-12

**Authors:** Li-fen Han, Jian-ming Zheng, Li-qing Zheng, Hai-bing Gao, Li-xia Chen, Qing-ling Xu, Yi-hong Chai, Xin Zhang, Chen Pan, Lv-feng Yao

**Affiliations:** 1 Department of Liver Diseases, Mengchao Hepatobiliary Hospital of Fujian Medical University, Fuzhou, 350025 China; 2 Department of Infectious Diseases, Huashan Hospital, Fudan University, Shanghai, 200040 China; 3 Department of Liver Diseases, The Second Hospital of Longyan, Fuzhou, 350025 China

**Keywords:** Telbivudine, Mother-to-child transmission, Chronic hepatitis B

## Abstract

**Background:**

To evaluate the efficacy and safety of telbivudine in chronic hepatitis B women during the second and third trimesters of pregnancy.

**Methods:**

The week 12–34 of pregnant women were screened in this prospective non-intervention study, with HBV DNA > 10^6^ IU/mL and alanine aminotransferase > 50 IU/L. The patients were received telbivudine treatment as a treatment group or without antiviral treatment as a control group. All infants were received recombinant hepatitis B vaccine 10 μg within 12 h of birth, at week 4 and week 24, immunoglobulin G within 12 h of birth and were detected HBV markers at the range from 7 to 12 months after delivery.

**Results:**

A total of 241 patients were finally enrolled, 139 patients in telbivudine group and 102 patients in control group. HBsAg negative rate of infants was 99.3% (135/136) in telbivudine group and was 91.9% (91/99) in control group after 7 months (*P* = 0.005), respectively. The incidence of undetectable HBV DNA levels (47.5%) was significantly lower in telbivudine-treated mothers than that in the controls (0%), and 75.5% patients alanine aminotransferase returned to normal in telbivudine group, and 51% in control group at delivery (*P* < 0.001), respectively.

**Conclusions:**

Telbivudine can safely reduce mother-to-child transmission in chronic hepatitis B women after 12 weeks of gestation.

## Background

Although the weighted prevalence of hepatitis B surface antigen (HBsAg) for Chinese population aged 1–59 years were 7.2%, immunization program should be further strengthened to reach those remaining at highest risk [1]. Perinatal or mother-to-child transmission (MTCT) is the most common form of transmission of hepatitis B virus (HBV) in many high-prevalence areas, particularly in Asian countries. The regions of the world where HBV genotype C is found as MTCT is associated with high maternal viral load (HBV DNA > 10^6^ IU/mL), and may occur in up to 90% of mothers who are HBsAg positive and hepatitis B e antigen (HBeAg) positive in the absence of prophylaxis [2–9]. Application of Hepatitis B vaccine combined with Hepatitis B immune globulin (HBIG) considerably reduced perinatal transmission [10]. However, in a previous study from China, the rate of immunoprophylaxis failure by predelivery HBV DNA levels, was 0% for levels < 10^6^ copies/mL, 3.2% for levels of 6–6.99 log_10_ copies/mL, 6.7% for levels between 7 and 7.99 log_10_ copies/mL, and 7.6% for levels > 8 log_10_ copies/mL, respectively [9]. No perinatal transmission has also been reported in infants born to mothers with viral loads < 6 log_10_ copies/mL in other studies [6, 11]. The HBV DNA threshold to consider antiviral therapy to prevent perinatal transmission is > 2 × 10^5^ IU/mL [9]. The use of these agents in HBeAg positive women whose HBV DNA are > 10^6^ copies/mL in the third trimester to prevent MTCT is recommended [12]. Thus, antiviral therapy was started at 28–32 weeks of gestation in most of the studies [13]. It’s still have failed immunoprophylaxis in some high viral load mothers, so antiviral therapy start at a earlier time may be considered such as at the second trimesters of pregnancy [14]. Since tenofovir is not approved for the treatment of chronic hepatitis B (CHB) before 2014 in China and very expensive, the use of telbivudine would appear to be a favorable treatment option for CHB mothers [15]. Moreover, some previous studies found that patients in the telbivudine group had significantly lower HBV DNA and HBeAg levels and higher HBV DNA negative conversion rates compared to those in the lamivudine group before delivery [16, 17]. Thus, we performed this study to evaluate the efficacy and safety of using telbivudine treatment started at 12–34 weeks of gestation in HBeAg positive or negative CHB mothers (rare of the studies including HBeAg negative mothers) on MTCT prevention, HBV DNA suppression, and maternal and fetal safety including major birth defect rates.

## Methods

### Patients

A total of 241 pregnant women with chronic hepatitis B treated from January 2012 to March 2015 at Mengchao Hepatobiliary Hospital of Fujian Medical University, were enrolled finally. Inclusion criteria were: (1) age between 18 and 45 years, (2) gestational age between 12 and 34 weeks, (3) a history of serum HBsAg positivity for more than 6 months, (4) serum HBV DNA > 10^6^ IU/mL, (5) serum alanine aminotransferase (ALT) above upper limit of normal (ULN) (50 IU/mL) and/or serum aspartate transaminase (AST) above ULN (40 IU/mL), (6) serum total bilirubin (TBIL) below 125 μmol/L. Exclusion criteria included: (1) liver failure or cirrhosis, (2) coinfection with hepatitis A, C, D or E, syphilis or human immunodeficiency virus, (3) with severe respiratory, circulatory, urinary or nervous system diseases, (4) a history of antiviral therapy, (5) clinical signs of threatened miscarriage in early time of pregnancy, (6) concurrent treatment with immune modulators, cytotoxic drugs, or steroids. Withdrawal criteria were: (1) poor compliance and lack of regular follow-up, (2) administration of other drugs, e.g. immunomodulators, cytotoxic drugs or steroids during the following time, (3) switch to other treatment (for example, patients in control group were received telbivudine or other antiviral drugs in this study). This prospective observational study was approved by the Ethics Committee of Mengchao Hepatobiliary Hospital of Fujian Medical University (award number 2011–001-01), and all the patients signed informed consent. All procedures were in accordance with the Helsinki Declaration of 1975. The written consent was obtained from every mother, and all subjects consented before screening for the study. The patients were assigned to the telbivudine group or without antiviral treatment groups according to their own preferences.

### Treatment

In our study, 139 patients were received telbivudine as telbivudine group and 102 patients without antiviral treatment as control group, according to their own preferences at the baseline. The telbivudine group were administered telbivudine (Sebivo, 600 mg/d, Beijing Novartis Pharma Ltd.) from 12 to 34 weeks of gestation. The patients in control group were not received any antiviral drug, but received agents for improving liver function. All of the patients in two groups with abnormal liver function were received glycyrrhizin (Minophagen Pharmaceutical Co., Ltd., Japan), Polyene Phosphatidylcholine (Essentiale, Sanofi Beijing Pharma Ltd.), ademetionine (Simeitai, Abbott Laboratories Ltd.) or other agents for improving liver function. The patients in telbivudine group continued to use telbivudine treatment after delivery.

All newborns were vaccinated with genetically engineered hepatitis B vaccine 10 μg in the deltoid muscle within 12 h of birth, at week 4 and 24 and HBIG 100–200 IU in the gluteus maximums within 12 h of birth. Due to the lack of safety data for breastfeeding with antiviral treatment, breastfeeding was not encouraged in our study and all newborns were bottle-fed in telbivudine group. Breast feeding could have been allowed for control group as this is not a blinded study.

### Outcomes assessments

The enrolled women received routine physical examination during pregnancy. The liver function, HBV markers, HBV DNA, Creatine kinase (CK), routine blood test and renal function were assessed regularly. Renal function and CK were monitored every 2–4 weeks in telbivudine group. Recently, new evidences recommended that infants who were seropositive for HBsAg and HBV DNA at 7 months could be identified as having acquired HBV infection [15, 18, 19]. Thus, serum HBV markers of infants was measured once at the time range from 7 to 12 months after delivery due to not all infants were able to be assessed at 7 months after delivery in our study. The liver function, renal function and CK were assessed on an Beckman Coulter Biochemistry Analyzer with corollary reagents. HBV serum markers were detected by the Abbott chemiluminescence assay based on ARCHITECT i2000SR. Serum HBV DNA levels were detected by fluorescence quantitative PCR (Shanghai Fosun Pharmaceutical Co., Ltd.) based on ABI7500, with a detection range from 500 IU/mL to10^9^ IU/mL. HBeAg conversion was defined that HBeAg was loss and Hepatitis B e antibody (HBeAb) was positive after antiviral treatment.

### Statistical analysis

Statistical analysis were performed using the SPSS19.0 (SPSS, Inc., Chicago, IL) software. Continuous variables were expressed as mean ± standard deviation (SD) unless otherwise specified, and t tests or non-parametric Mann-Whitney U-test were used for comparing differences between two groups if necessary. For categorical variables, the chi-square test or Fisher exact test was used for group comparisons. Significance level was set at *P* < 0.05.

## Results

### Baseline characteristics

**Fig. 1:**
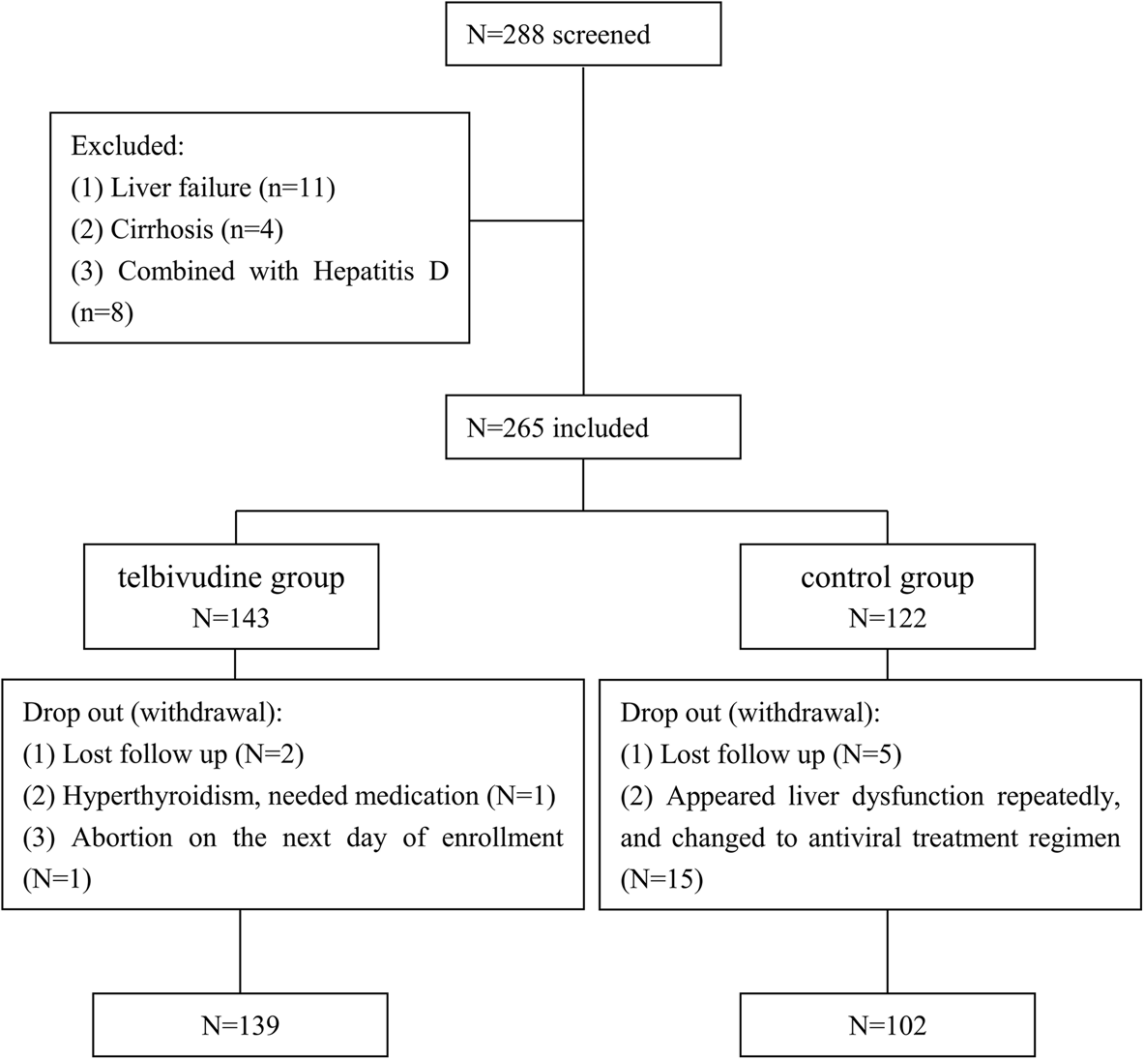
Flowchart for patients enrollment. A total of 288 at 12–34 weeks of gestation women with chronic hepatitis B were screened, 23 patients did not meet the eligibility criteria and were excluded. In the remaining 265 individuals, there were 143 patients in telbivudine group and 122 patients in control group. However, 4 patients and 20 patients withdrew from the study in each group, respectively. Therefore, there were 139 patients in telbivudine group and 102 patients in control group in our study cohort finally

A total of 288 at 12–34 weeks of gestation women with chronic hepatitis B were screened, 23 patients did not meet the eligibility criteria and were excluded. In the remaining 265 individuals, there were 143 patients in telbivudine group and 122 patients in control group. However, 4 patients and 20 patients withdrew from the study in each group, respectively. Therefore, there were 139 patients in telbivudine group and 102 patients in control group in our study cohort finally (Fig. 1). In telbivudine group, three patients started antiviral treatment at 12 weeks, 1 patient started at 13 weeks, 5 patients started at 14 weeks, 5 patients started at 15 weeks, 4 patients started at 16 weeks, 2 patients started at 17 weeks, 8 patients started at 18 weeks, 5 patients started at 19 weeks, 4 patients started at 20 weeks, 9 patients started at 21 weeks, 5 patients started at 22 weeks, 7 patients started at 23 weeks, 2 patients started at 24 weeks, 6 patients started at 25 weeks, 9 patients started at 26 weeks, 4 patients started at 27 weeks, 18 patients started at 28 weeks, 8 patients started at 29 weeks, 8 patients started at 30 weeks, 6 patients started at 31 weeks, 8 patients started at 32 weeks, 5 patients started at 33 weeks, 7 patients started at 34 weeks, respectively. In telbivudine group, 57.6% (80/139) patients started antiviral therapy at week 12–27 of gestation. Baseline characteristics of mothers was summarized in the Table 1.

**Table 1:** Baseline characteristics of mothers

Characteristics of mothers	Telbivudine group (*N* = 139)	Control group (*N* = 102)	*P* value
Age, years, median (range)	26 (20–43)	26 (18–42)	0.061
Prior pregnancy, median (range)	1 (1–4)	1 (1–4)	0.224
HBV DNA levels, log_10_ IU/mL, median (range)	7.73 (6.04~9.30)	7.72 (6.03~9.00)	0.858
HBV DNA levels > 10^8^ IU/mL, n (%)	60 (43.2%)	45 (44.1%)	0.883
HBV DNA levels 10^7^–10^8^ IU/mL, n (%)	62 (44.6%)	36 (35.3%)	0.184
ALT level, U/L, median (range)	117 (56–1166)	164 (53–1025)	0.867
AST level, U/L, median (range)	112 (32–1061)	101 (35–539)	0.301
Elevated AST levels, n (%)	136 (97.8%)	101 (99.0%)	0.640
TBIL, μmol/L, median (range)	11 (4–125)	11 (3–96)	0.911
HBeAg positive, n (%)	126 (90.6%)	91 (89.2%)	0.714

### Efficacy of telbivudine treatment

**Fig. 2:**
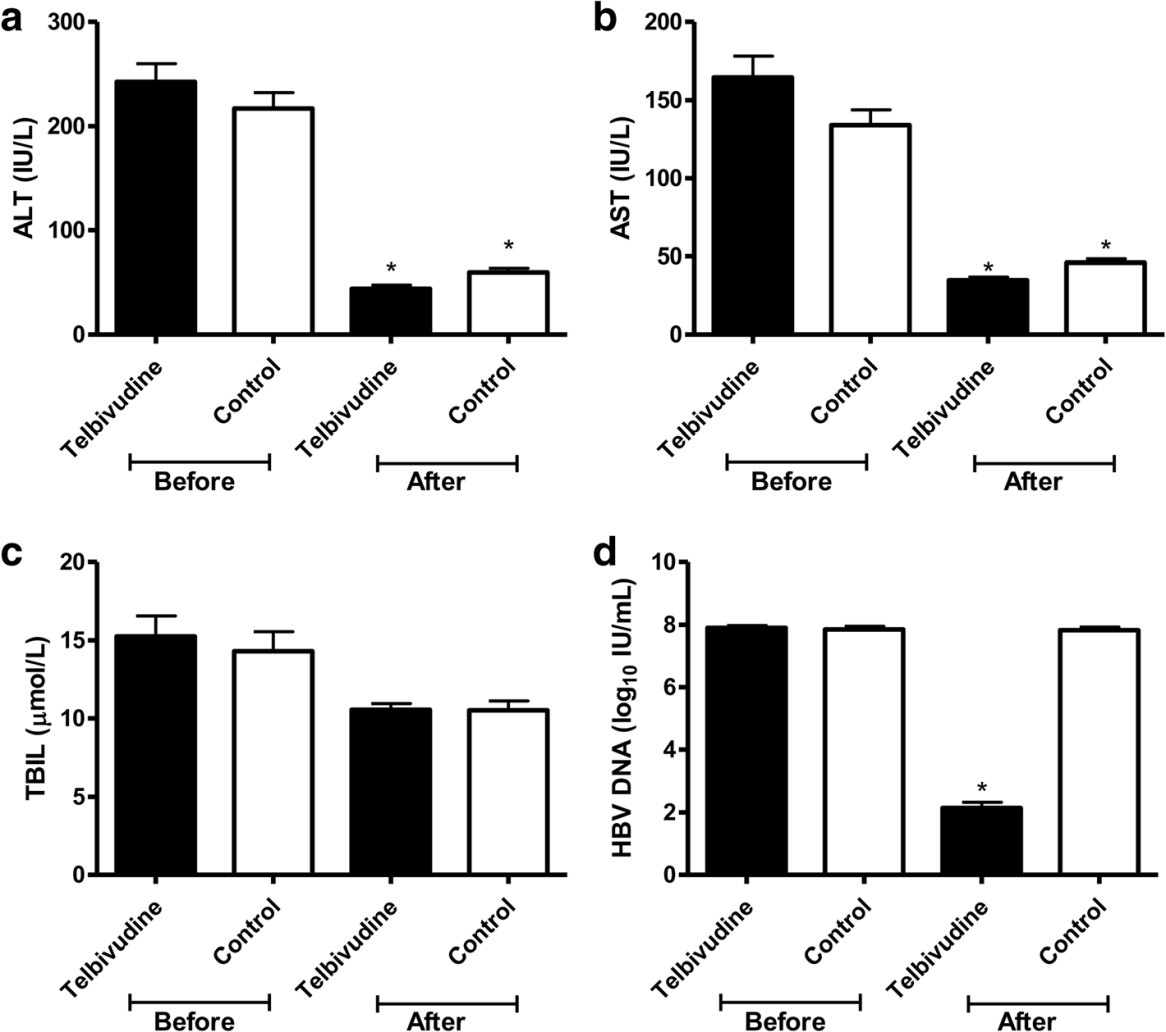
ALT, AST, TBIL and HBV DNA levels at baseline and at delivery. Panel **a** was ALT, panel **b** was AST, panel **c** was TBIL, and panel **d** was HBV DNA.* means *P* < 0.05, compared with that before the treatment in the same drug group

**Fig. 3:**
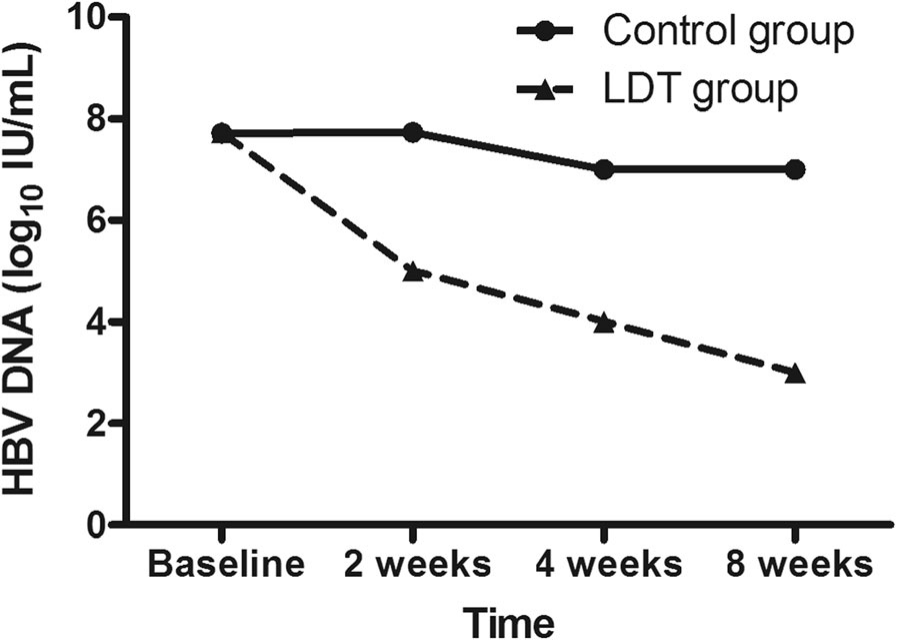
HBV DNA levels in different groups. There was no statistically significant difference between HBV DNA levels in telbivudine group and that in control group at baseline. HBV DNA levels at 2 weeks, 4 weeks and 8 weeks in telbivudine group was lower than that at the same time in control group (*P* < 0.0001)

At delivery, 75.5% (105/139) of patients ALT returned to normal in telbivudine group, compared to 51% (52/102) of patients in the control group (χ^2^ = 15.627, *P* < 0.001). AST in telbivudine group was lower than that in control group (χ^2^ = 19.643, *P* < 0.001), but TBIL was no different between two group (Table 2 and Fig. 2). In telbivudine group, the patients were received telbivudine for an average of 13 ± 6 weeks, and HBV DNA levels was declined to 2.82 log_10_ IU/mL at delivery, compared to the baseline (U = 13, *P* < 0.001). HBV DNA level was declined after telbivudine treatment, but it didn’t decline in control group (Fig. 3). There was no statistically significant difference between HBV DNA levels at baseline and that at delivery in control group (Fig. 2). At delivery, HBV DNA levels in telbivudine group was lower than that in control group (U = 28, *P* < 0.001). Furthermore, HBV DNA levels of 47.5% (66/139) patients were undetectable at delivery in telbivudine group, but none of the patients HBV DNA levels was undetectable in the control group (*P* < 0.001). All patients were follow-up for 6 months after delivery. The patients in telbivudine group continued to use telbivudine treatment after delivery, but five patients stopped telbivudine treatment after delivery. HBV DNA levels of one patient who stopped telbivudine, increased up to 8 log_10_ IU/mL 2 months later, then ALT was 433 U/L and AST was 160 U/L 3 months later. This patient’s liver function returned to normal again after 2 months antiviral treatment with Pegasys and entecavir. The liver function of other patients remained normal, who stopped antiviral treatment, but HBV DNA levels were ranged from 5.00 log_10_ IU/mL to 8.64 log_10_ IU/mL. None of them had virological breakthrough, who continued to use telbivudine therapy. Among the 126 HBeAg positive mothers in telbivudine group, 14.3% (18/126) patients had HBeAg seroconversion at delivery. However, none of patients (91 HBeAg positive patients) had HBeAg seroconversion in control group (*P* < 0.001).

**Table 2:** Efficacy of telbivudine treatment

	Telbivudine (*N* = 139)	Control (*N* = 102)	*P* value
HBV DNA, log_10_ IU/mL, median (range)			
Week 2	5.51 (3.04–8.06)	7.74 (6.03–9.00)	< 0.001
Week 4	4.12 (2.70–7.43)	7.70 (6.03–9.00)	< 0.001
Week 8	3.20 (2.70–6.76)	7.74 (6.03–9.00)	< 0.001
At delivery	2.82 (2.70–6.45)	7.72 (5.32–9.00)	< 0.001
ALT levels at delivery, U/L, median (range)	28 (11–238)	47 (8–217)	< 0.001
Normalization rate of ALT, n (%)	105 (75.5%)	52 (51%)	< 0.001
AST levels at delivery, U/L, median (range)	27 (11–141)	39 (17–144)	< 0.001
Normalization rate of AST, n (%)	112 (80.6%)	55 (53.9%)	< 0.001
TBIL levels at delivery, U/L, median (range)	1.1 (9.8–36.0)	9.5 (3.8–48.6)	0.459
Normalization rate of TBIL, n (%)	137 (98.6%)	100 (98.0%)	1.000
HBeAg positive at delivery, n (%)	108 (77.7%)	91 (89.2%)	0.025

Finally, 137 infants were born in telbivudine group, 99 infants were in control group (*P* = 1.0), respectively. All newborns were vaccinated with genetically engineered hepatitis B vaccine 10 μg in the deltoid muscle within 12 h of birth, at week 4 and 24 and HBIG 100–200 IU in the gluteus maximums within 12 h of birth. HBsAg was screened in 235 infants at the age ranged from 7 to 12 months, due to one infant died of ruptured abdominal aortic aneurysm at 8 days after delivery in telbivudine group. HBsAg negative rate of infants was 99.3% (135/136) in telbivudine group, and was 91.9% (91/99) in control group (*P* = 0.005), respectively. The mother of HBsAg positive infant in telbivudine group, whose HBeAg was positive, had received telbivudine treatment since week 34 of gestation and her serum HBV DNA levels was 6.15 log_10_ IU/mL at delivery. In control group, the HBV DNA levels of patients who had HBsAg positive infant was 8.33 ± 0.88 log_10_ IU/mL, compare with that of patients had HBsAg negative infant was 7.69 (median, ranged from 5.32–9.00) log_10_ IU/mL (U = 259, *P* = 0.141). In control group, 11 HBeAg negative mothers had 11 infants and only one infant’s HBsAg was positive. HBsAg negative rate of infants in HBeAg negative mothers in control group was no significant difference compared with HBeAg positive mothers in control group (*P* = 1.000). HBsAg negative rate of infants in HBeAg negative mothers in telbivudine group was also no significant difference compared with HBeAg positive mothers in telbivudine group(*P* = 0.458). The liver function of HBsAg positive infants was normal, so they were planned to follow up every 6 month.

### Safety of telbivudine treatment

Creatine kinase of 7 patients slightly increased in the telbivudine group after antiviral treatment, and the maximum value was 367 U/L. None of them presented obvious symptoms. CK did not increase in any patient of control group, and was statistically significant difference compared with that of telbivudine group (*P* = 0.022). The rate of adverse events was 4.3% (6/139) in telbivudine group, and was 5.9% (6/102) in control group (χ^2^ = 0.305, *P* = 0.581), respectively (Table 3).

**Table 3:** Adverse events reported in this study

	Telbivudine	Control	*P* value
CK Elevation of mother, n (%)	7 (5%)	0 (0%)	0.022
Malformation induced labor, n	1	1	1.000
Abortion, n	1	2	0.575
Premature labor, n	3	2	1.000
Congenital diseases, n	1	1	1.000

The median of gestational age was 38 weeks in telbivudine group, and 38 weeks in control group (U = 6217, *P* = 0.085), respectively. The rate of cesarean section was 35% in telbivudine group, and 36% in control group (*P* = 0.892), respectively. The rates of cesarean section were high in this study, but consistent with the rate of cesarean section observed in China [20]. In telbivudine group, one infant had abdominal aortic aneurysm. In control group, one infant had cleft lip. However, there no significant difference on birth defect between two groups (*P* = 1.000) (Table 3).

## Discussion

According to national guidelines 2010 version, application of Hepatitis B vaccine combined with HBIG is as the standard of care for prevent MTCT of HBV in China. By the end of 2015, the use of lamivudine, telbivudine or tenofovir in HBeAg positive women whose HBV DNA are > 10^6^ copies/mL at 28–32 weeks of gestation is added as a new method in Chinese guide 2015 version to prevent MTCT [21, 22]. In our study, antiviral therapy was started at week 12–34 of pregnancy, some of them started antiviral therapy earlier than previous studies, which antiviral therapy was started at week 28–32 of pregnancy according to the American Association for the Study of Liver Disease (AASLD) guideline [13]. In the telbivudine group, only one infant’s HBsAg was positive after 7 months, whose mother started antiviral therapy at 34 weeks of gestation. It maybe too later, so her HBV DNA levels was still above 6 log_10_ IU/mL at delivery. If the patient who started antiviral therapy at 34 weeks of pregnancy was not included in our study, the rate of immunoprophylaxis failure was zero. That is very encouraging and impressive. About 57.6% patients started antiviral therapy at week 12–27 of gestation, but adverse events reported in telbivudine group was similar to control group.

In our study, we enrolled the patients who started antiviral treatment at ALT above > one fold the upper limit of normal, which is lower than AASLD or Asian Pacific Association for the Study of the Liver (APASL) guideline, but the same as the European Association for the Study of the Liver (EASL) guideline [13, 23, 24]. Thus, more people may achieve benefit from our study.

Treatment was usually administered in the second and third trimesters of gestation, because the most sensitive period of fetal development generally occurs in the first trimester, especially at the first 8 weeks of pregnancy [25]. Telbivudine is an L-reverse transcriptase inhibitor, and has shown no effects on human nucleotides or DNA synthesis [26]. Toxicological research has demonstrated that telbivudine has no carcinogenic, teratogenic, or mutagenic effects, and no mitochondrial toxicity [26, 27]. The duration of antiviral treatment should be considered, because it requires several weeks to suppress serum viral loads effectively. It would be desirable to minimize the adverse events to fetal development while to suppress serum HBV DNA levels of pregnant women as early as possible, so that it can probably enhance the efficacy of the antiviral agent in reducing MTCT of HBV. Therefore, we evaluated the patients received antiviral treatment at 12–34 weeks of gestation more earlier than previous studies, and the limited safety data suggest that it did not increase the risks of adverse events of maternal or fetal. The results of our study was similar to two previous studies, which the initiation of NA treatment time were at week 12–30 and week 8–32, but those studies were only enrolled HBeAg positive women with chronic hepatitis B [16, 28]. Moreover, another previous study shown that treatment with telbivudine during the second and third trimesters of pregnancy safely blocked perinatal transmission of HBV and infants born to telbivudine-treated mothers showed normal growth and development during long-term follow-up of up to 5 years [14].

The most common telbivudine-related adverse events was increased serum CK levels [29]. In our study, CK levels was slightly increased in 7 cases from telbivudine group and spontaneously recovered without drug withdrawal. In telbivudine group, fetal malformation was found in two cases (1 case at antenatal examination after 4 weeks of treatment, and another newborn had a ruptured abdominal aortic aneurysm in the perinatal period), and it’s no statistically significant difference compared with that in control group. These findings indicated that telbivudine treatment is safe in the second and third trimesters. Another study of 489 pregnancies found that treatment with telbivudine presents a favorable safety profile without increasing the rates of live birth defect, spontaneous abortion or elective termination, and neonatal toxicity [30].

There were a few limitations of this study. First, there maybe have a risk of bias in this single center and observational study. Second, all the patients were naïve treatment patients with serum HBV DNA > 6.0 log_10_ IU/mL, the efficacy of telbivudine in the CHB patients with a history of antiviral therapy on MTCT was still unknown. Third, we didn’t detect genotypes of HBV in this study, which was a important point for antiviral treatment. However, viral genotypes have diverse geographical distribution and the genotypes of HBV are almost genotype B or C in China according to a previous study [31].

## Conclusions

The antiviral therapy in HBeAg positive women whose HBV DNA are > 10^6^ IU/mL was started at the third trimester to prevent MTCT is recommended. However, it’s still have failed immunoprophylaxis in some high viral load CHB mothers. Antiviral therapy start at a earlier time may be a solution, but we have insufficient data to prove that it is a safe method. In this study, we found that telbivudine treatment at 12–34 weeks of gestation in HBeAg positive or negative CHB mothers with serum HBV DNA > 6.0 log_10_ IU/mL is well tolerance and safe in our limited data. Thus, telbivudine can safely reduce mother-to-child transmission in chronic hepatitis B women after 12 weeks of gestation.

## Data Availability

Data supporting our findings is contained within the manuscript. Data is available from the corresponding author upon request. Identifying/confidential patient data however will not be shared.

## Abbreviations

AASLD: American Association for the Study of Liver Disease
ALT: Alanine aminotransferase
APASL: Asian Pacific Association for the Study of the Liver
AST: Aspartate transaminase
CHB: Chronic hepatitis B
CK: Creatine kinase
EASL: European Association for the Study of the Liver
HBeAg: Hepatitis B e antigen
HBIG: Hepatitis B immune globulin
HBsAg: Hepatitis B surface antigen
HBV: Hepatitis B virus
MTCT: Mother-to-child transmission
TBIL: Total bilirubin
ULN: Upper limit of normal
